# Supplementary material for: Paradoxical neuronal hyperexcitability in a mouse model of mitochondrial pyruvate import deficiency
Source: eLife. 2022 Feb 21;11:e72595. doi: 10.7554/eLife.72595 (PMC8860443; doi:10.7554/eLife.72595)
Supplement: Source data 1. [file elife-72595-data1.pptx]

## Slide 1
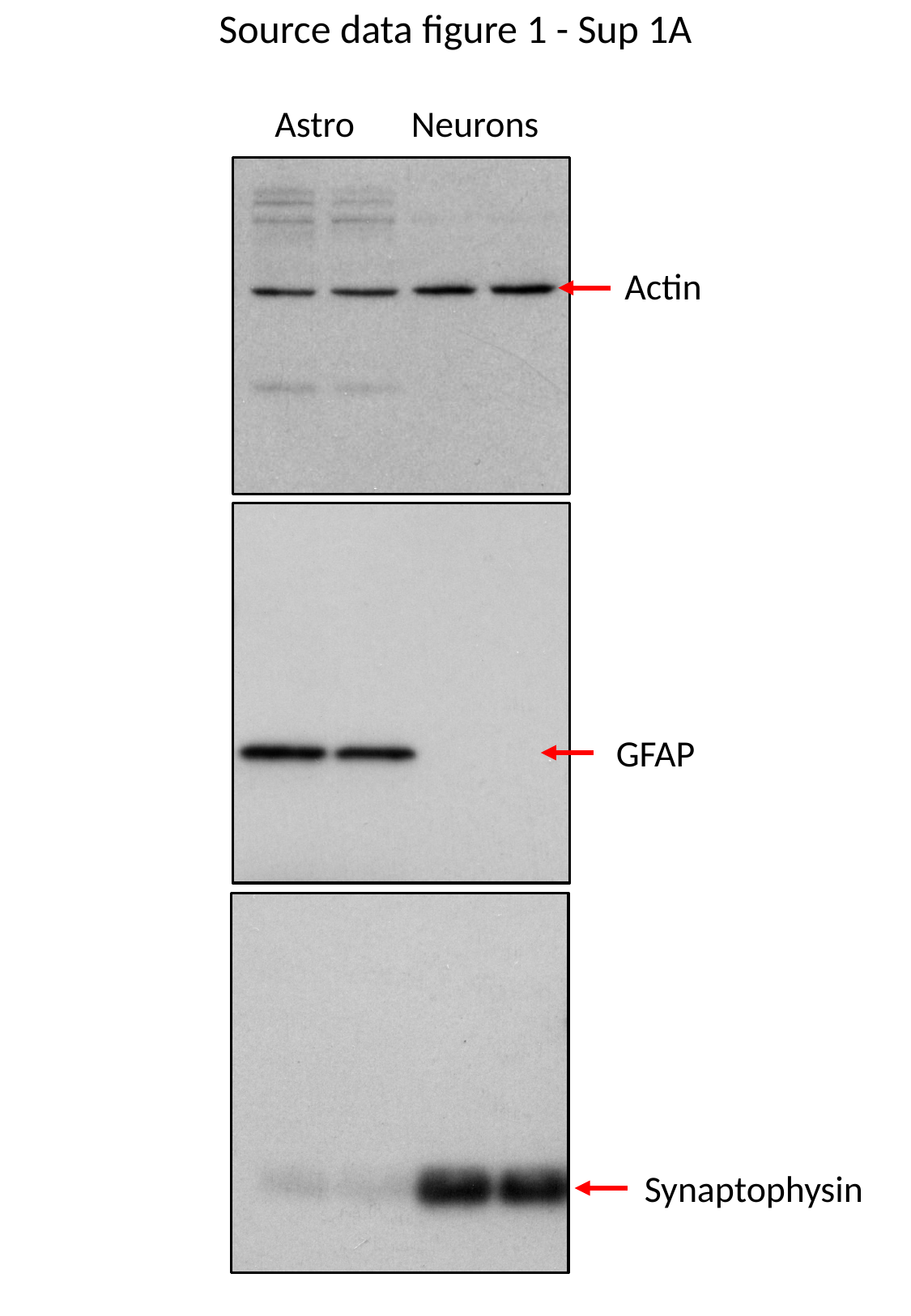

Source data figure 1 - Sup 1A
Astro
Neurons
Actin
GFAP
Synaptophysin

## Slide 2
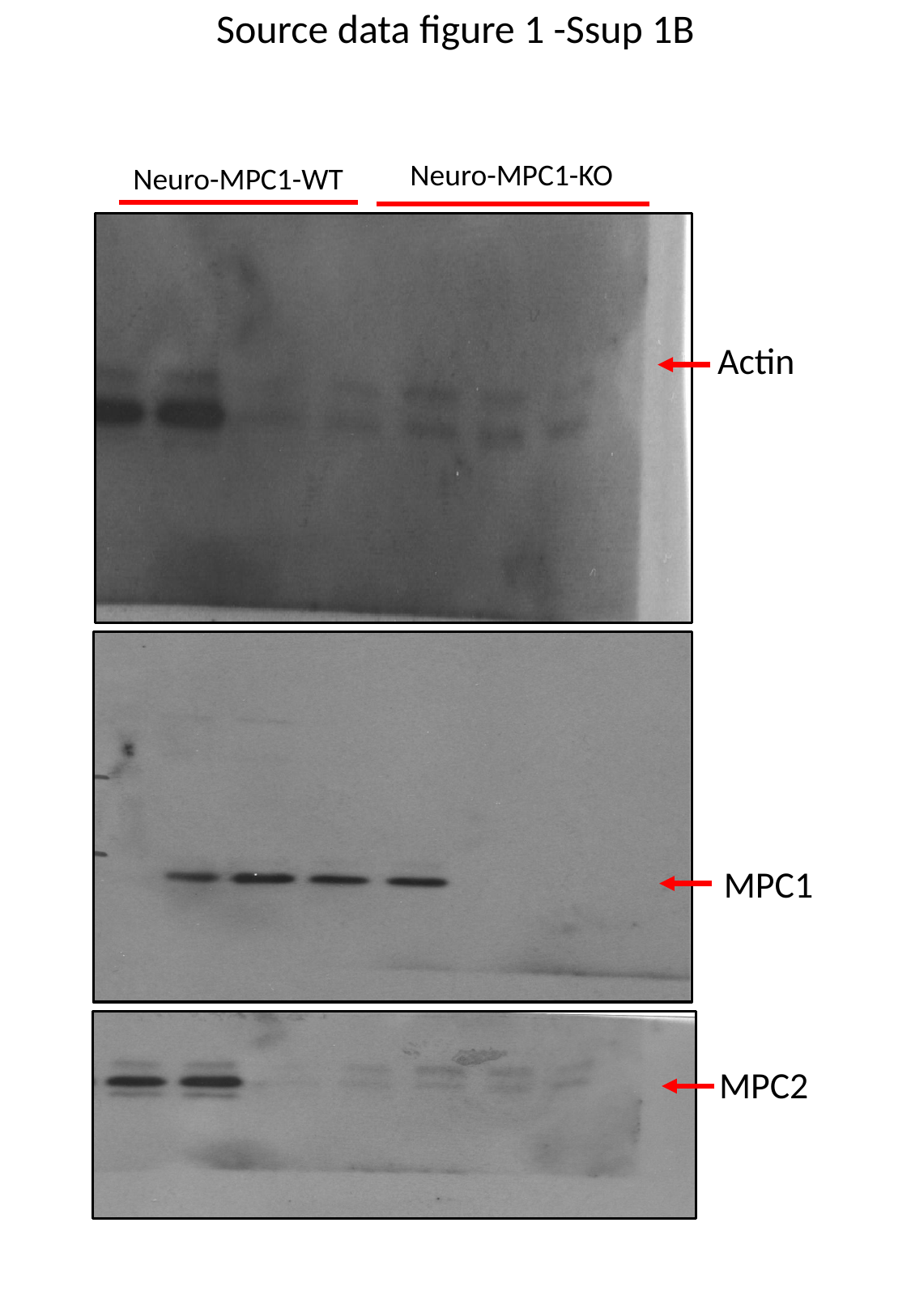

Source data figure 1 -Ssup 1B
Neuro-MPC1-KO
Neuro-MPC1-WT
Actin
MPC1
MPC2

## Slide 3
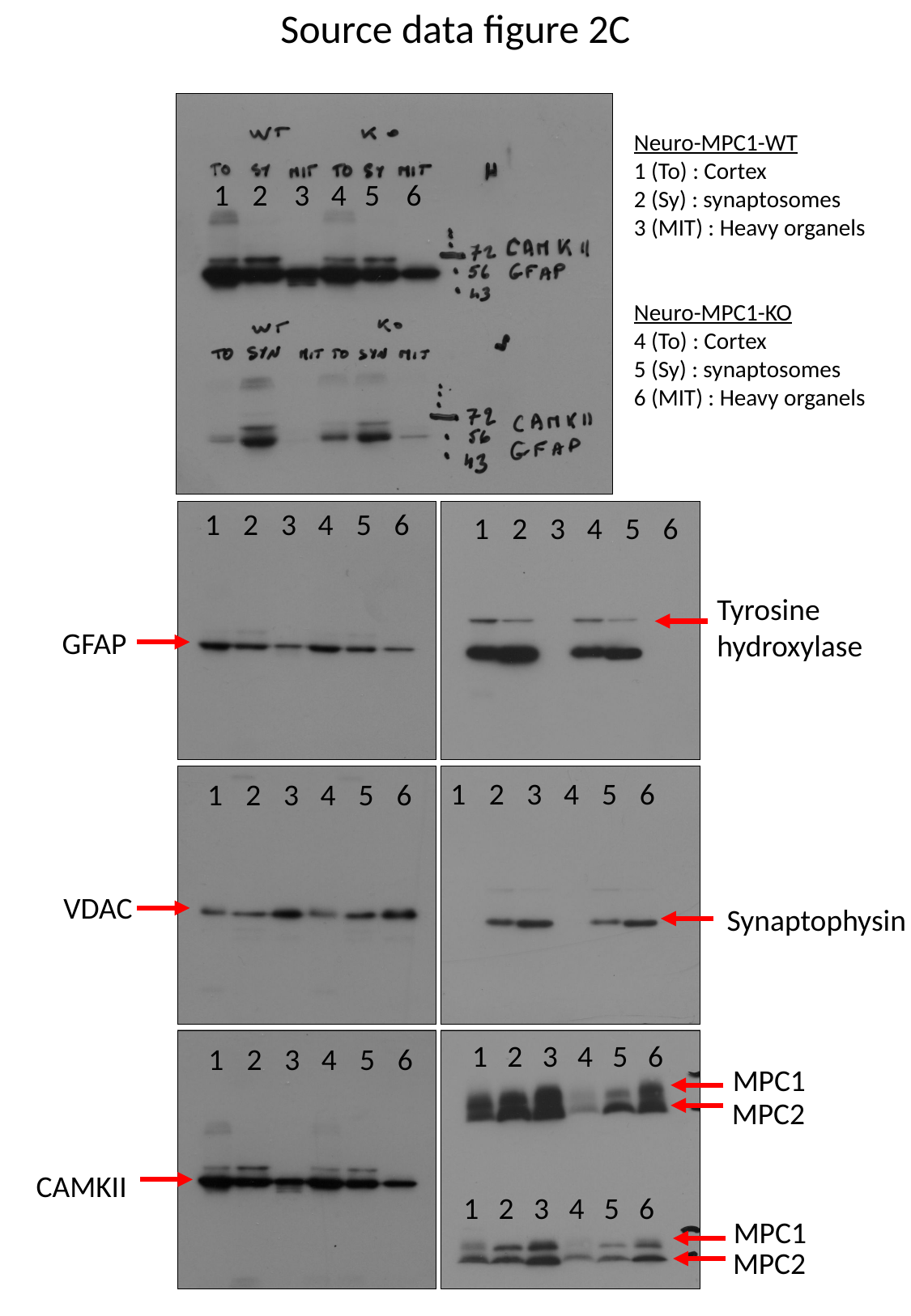

Source data figure 2C
Neuro-MPC1-WT
1 (To) : Cortex
2 (Sy) : synaptosomes
3 (MIT) : Heavy organels
Neuro-MPC1-KO
4 (To) : Cortex
5 (Sy) : synaptosomes
6 (MIT) : Heavy organels
1
2
3
4
5
6
1
2
3
4
5
6
1
2
3
4
5
6
Tyrosine hydroxylase
GFAP
1
2
3
4
5
6
1
2
3
4
5
6
VDAC
Synaptophysin
1
2
3
4
5
6
1
2
3
4
5
6
MPC1
MPC2
CAMKII
1
2
3
4
5
6
MPC1
MPC2

## Slide 4
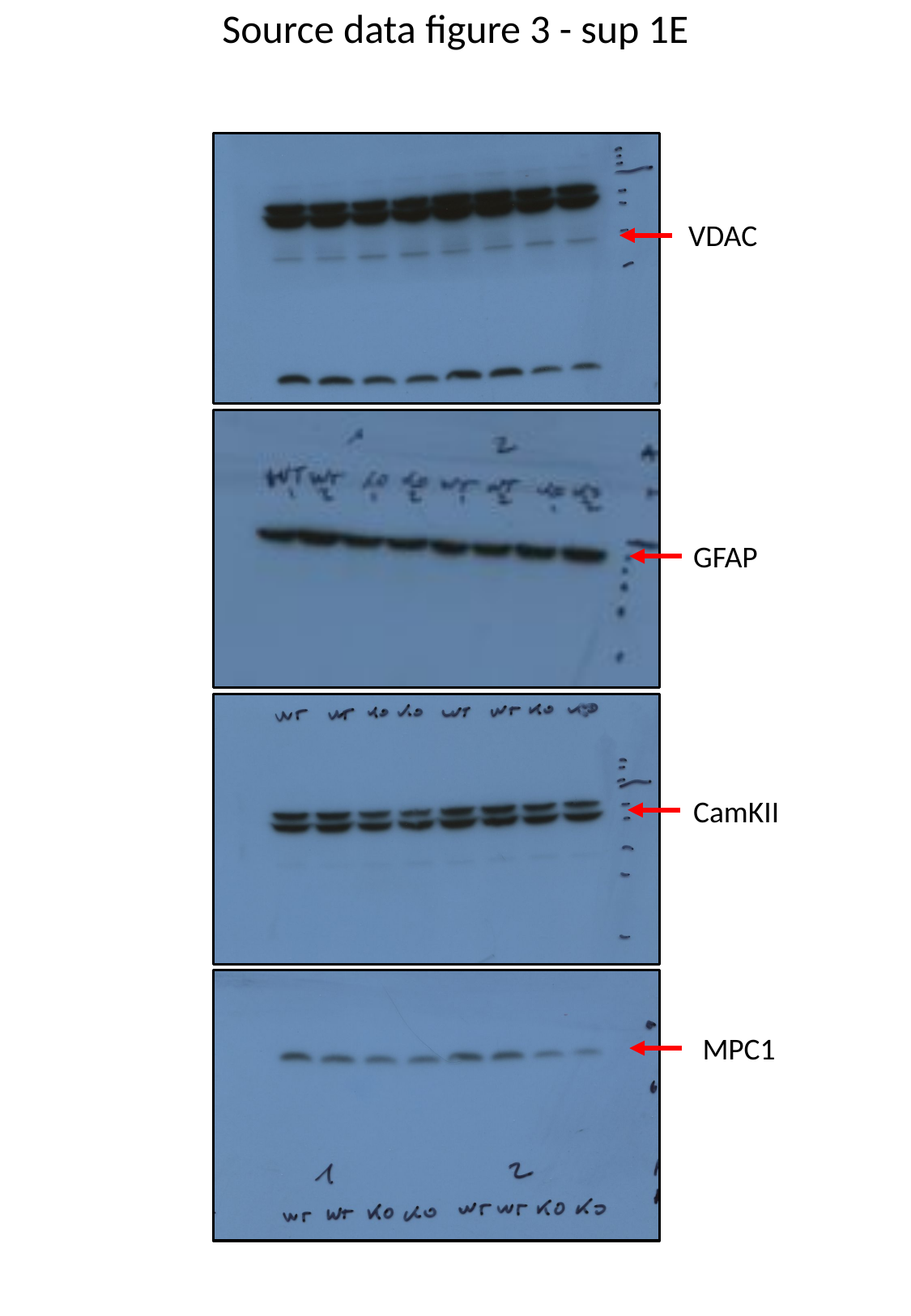

Source data figure 3 - sup 1E
VDAC
GFAP
CamKII
MPC1

## Slide 5
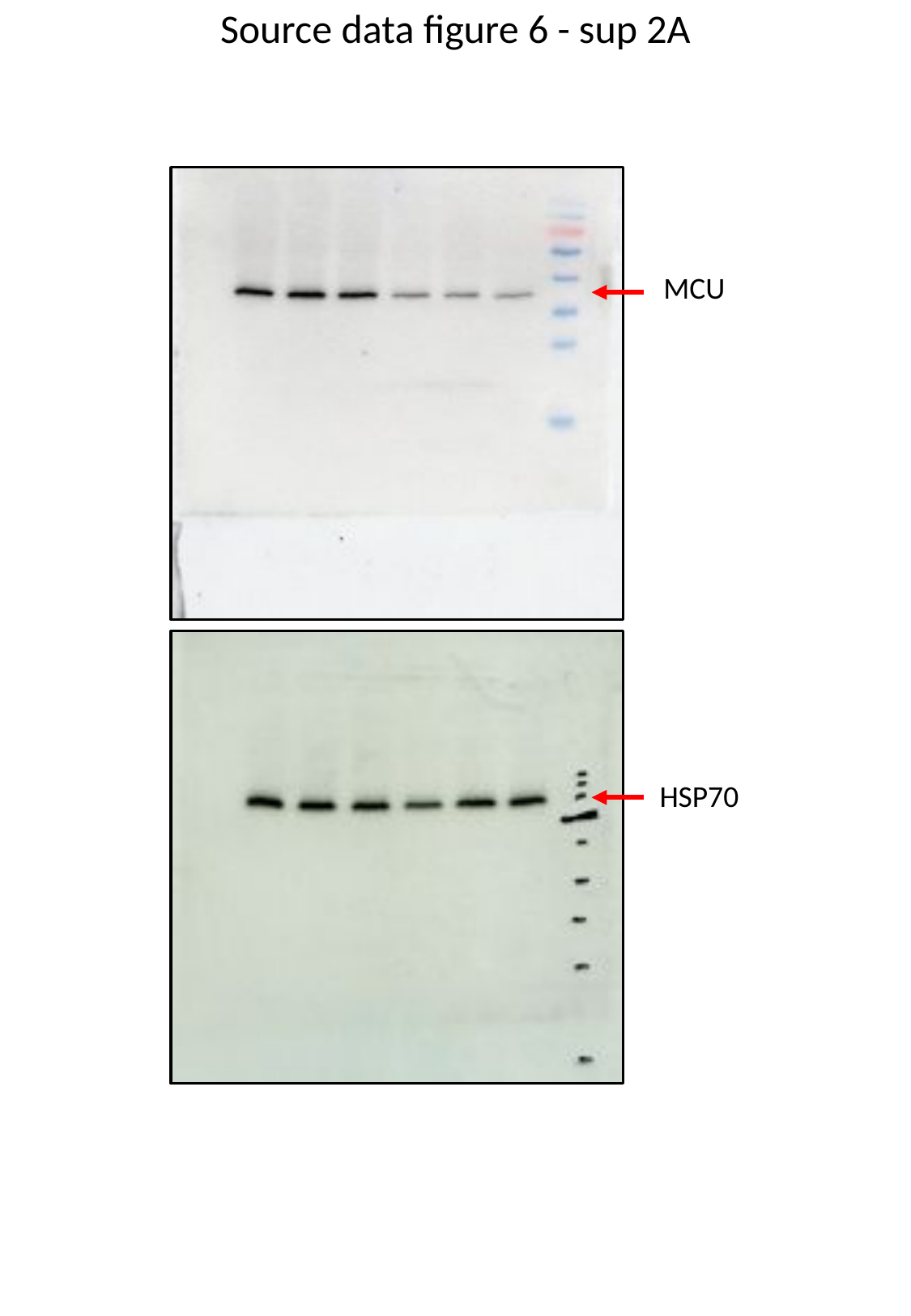

Source data figure 6 - sup 2A
MCU
HSP70
